# Redescription of *Aspidogaster limacoides* Diesing, 1834 (Aspidogastrea: Aspidogastridae) from freshwater fishes of northern Germany

**DOI:** 10.1007/s00436-021-07253-1

**Published:** 2021-08-25

**Authors:** Jaydipbhai Suthar, Sarah Al-Jufaili, Rodney A. Bray, Marcus Frank, Stefan Theisen, Harry W. Palm

**Affiliations:** 1 Aquaculture and Sea-Ranching, Faculty of Agricultural and Environmental Sciences, University of Rostock, Rostock, Germany; 2 Laboratory of Microbiology Analysis, Fishery Quality Control Center, Ministry of Agriculture, Fisheries Wealth and Water Resources, Al Bustan, Muscat, Sultanate of Oman; 3 Department of Life Sciences, Natural History Museum, London, UK; 4 Medical Biology and Electron Microscopy Centre, University Medicine, University of Rostock, Rostock, Germany; 5 Department Life, Light and Matter, University of Rostock, Rostock, Germany

**Keywords:** Trematoda, Taxonomy, Phylogeny, Cyprinidae

## Abstract

*Aspidogaster limacoides* Diesing, 1834 (Aspidogastridae) is redescribed based on light and scanning electron microscopy of specimens from the stomach and intestine of *Abramis brama*, *Rutilus rutilus* and *Scardinius erythrophthalmus* (Actinopterygii: Cyprinidae). The fishes were sampled during 2018 and 2019 at Lake Tollense in Mecklenburg-Western Pomerania, Germany. The prevalence of *A. limacoides* was highest in *R. rutilus* (61.7%) followed by *Scardinius erythrophthalmus* (7.7%) and *A. brama* (2.9%), while it was absent in *Perca fluviatilis* from the same lake. The following structures of *A. limacoides* are described for the first time: a depression on the ventral side of the neck, variations in the number and the arrangement of alveoli, numerous pits scattered all over the body surface, the presence of a few papillae-like structures posterior lateral to the mouth, the number of marginal organs represented by openings of exocrine multicellular glands as shown in histology and the subterminal position of the excretory pore. These characters can be used to distinguish three species of *Aspidogaster*, namely, *A. ijimai*, *A. conchicola* and *A. limacoides*, suggesting that SEM is a useful and promising tool in differentiating *Aspidogaster* species. Comparison of molecular data of the ITS1-5.8S-ITS2 regions showed a 94% similarity to *A. limacoides* from the European part of Russia. Phylogenetic analysis showed that the present specimens clustered in the same clade with *A. limacoides* sensu stricto, forming a distinct group to the exclusion of congeners.

## Introduction

Members of the trematode subclass Aspidogastrea are characterized by a large, alveolated ventral disc and parasitize aquatic ectotherm animals. The subclass Aspidogastrea Faust and Tang, 1936 is an ancient small group of Platyhelminthes which includes only four families (Rohde 2002). Aspidogastreans infect molluscs, fish and reptiles and rarely crustaceans, in marine as well as in freshwater environments with 61 valid species (Alves et al. 2015). This group is sister to the Digenea, together forming the Trematoda, and is thought to retain some primitive features such as a simple life cycle. On the other hand, they have a much more complex nervous system and sensory receptors compared with other groups of Platyhelminthes (Rohde 1994). So far, these parasitic flatworms have not been reported to cause any disease in humans nor have been found to be a significant problem for aquaculture, fisheries and other seafood industries (Lee et al. 2017). However, *Aspidogaster conchicola* Baer, 1826 and *A. limacoides* have been reported to cause pathological problems in clams and fish (*Rutilus frisii*), respectively (Pauley and Becker 1968; Rahanandeh et al. 2016).

In total, 12 species of the genus *Aspidogaster* Baer, 1826 have been reported so far and, among them, three species in Europe: *Aspidogaster antipai* Lepsi, 1932, *A. conchicola* Baer, 1826 and *A. limacoides* Diesing, 1834 (Alves et al. 2015). *Aspidogaster limacoides* was reported in Germany from the river Weser by Reimer (2002) from *Rutilus rutilus* nearly two decades ago. A few years later, this species was recorded from neighbouring countries such as Austria and Poland from *Barbus barbus* and *Rutilus rutilus*, respectively (Popiołek et al. 2007; Schludermann et al. 2005). However, the morphological and taxonomical descriptions of this species were solely based on light microscopy, not applying scanning electron microscopy (SEM) combined with DNA sequencing data based phylogenetic techniques. Detailed studies on the members of the genus *Aspidogaster* Baer, 1826 including SEM are scarce and focused on only two species, namely, *A. ijimai* Kawamura, 1913 and *A. conchicola* Baer, 1826 (Gao et al. 2003; Halton and Lyness 1971). *Aspidogaster ijimai* was recently studied using SEM by Lee et al. (2017).

The objectives of this study are to provide a detailed light and SEM investigation of *A. limacoides* Diesing, 1834 from a northern location in Germany, including molecular and phylogenetic analyses of the sampled specimens.

## Materials and methods

### Host examination and parasite collection

In total, 181 specimens of roach *Rutilus rutilus* (*n* = 47), rudd *Scardinius erythrophthalmus* (*n* = 13), bream *Abramis brama* (*n* = 35) and perch *Perca fluviatilis* (*n* = 86) were sampled from Lake Tollense in the northern part of Germany during 2018–2019. The fishes were caught by local fishermen with gillnets, killed on site, locally deep frozen and kept on ice during transportation until arrival at the University of Rostock, Germany, where they were transferred to a freezer (− 18 °C) for storage until subsequent examination for parasites. According to standard protocols, the stomach and intestines of the fish were examined for parasites after recording the total and standard length and slaughter weight. The parasites were counted to obtain prevalence, intensity and abundance data as defined by Bush et al. (1997). Selected parasites were fixed with 70% EtOH and 4% formalin for microscopic examination; others were fixed and stored in 99.6% EtOH for further DNA analyses.

### Parasite preparation for examination

#### Light microscopy and histology

Selected specimens fixed with 70% EtOH were stained with acetic carmine for light microscopic examination. Specimens were dehydrated in an ascending series of ethanol (70%, 90% and twice in 100% ethanol), cleared in 50% eugenol followed by 100% eugenol and mounted in Canada balsam. The body, organs and taxonomically relevant characters were measured with measurements given in micrometres. Voucher specimens were deposited in the Natural History Museum Berlin (Museum für Naturkunde Berlin (ZMB), Germany).

Additional specimens were preserved in 4% formalin for histology. Following dehydration of the parasites in an ascending series of ethanol [70%, 80%, 90% and 100% (two times)], samples were placed into xylol (three times for 1 h) and then in paraffin at 59–60 °C for 1 h. For paraffin embedding, the samples were transferred into standard cassettes with paraffin. The paraffin slices containing parasites were cut into different sizes (5 µm, 10 µm and 20 µm) by using a rotation microtome (Leica RM 2255) at room temperature and placed in a water bath (Leica HI 1210). The paraffin slices were transferred onto slides and dried on a heating plate (Medax 14,800) at 37 °C, and the slides were placed in a rack and incubated overnight at 37–38 °C. For staining, the slides were dipped into xylol (three times for 10 min) for deparaffinizing, rehydrated in alcohol (100% two times for 1 min; 90%, 80%, 70% and 50% for one time for 1 min), deionized with water (three times for 2 min each time) and stained with haematoxylin and eosin and alcian blue (1% in 3% acetic acid) according to the standard staining protocols of the Institute for Anatomy, University Medicine, University of Rostock, dehydrated and cleared with an ethanol series and xylol. The slides were covered with mounting medium and a cover glass.

#### Scanning electron microscopy

For scanning electron microscopy, formalin-fixed specimens were dehydrated in an ascending ethanol series and transferred to 100% acetone (twice for 10 min each), critical point dried (Emitech K850, Co. Quorum Technologies LTD, East Sussex), mounted on SEM-carrier with adhesive conductive carbon tape (Co. PLANO, Wetzlar), coated with gold under vacuum (EM SCD 004, Co. BALTEC, Balzers) and analysed by a field emission scanning electron microscope (FE-SEM, MERLIN® VP Compact, Co. Zeiss, Oberkochen) at the Electron Microscopy Centre, University Medicine Rostock.

### DNA isolation, amplification and sequencing

Genomic DNA was extracted from mature worms stored in 99.6% EtOH following the standard protocol of the DNeasy Blood and Tissue kit (Qiagen, Hilden, Germany). The ribosomal DNA (rDNA) region comprising ITS-1, 5.8S, ITS-2 and flanking sequences (= ITS +) was amplified with a polymerase chain reaction (PCR) with the universal primers BD1 (5′-GTCGTAACAAGGTTTCCGTA-3′) and BD2 (5′-TATGCTTAA(G/A)TTCAGCGGGT-3′) (Luton et al. 1992). The PCR reaction was performed in a total volume of 50 µl consisting of 2.5 µl of each primer (10 pmol µl^−1^), 5 µl extracted DNA, 5 µl H_2_O and 25 µl ready-to-use master mix (Qiagen, Hilden, Germany). The amplification cycle consisted of the following conditions: initial denaturation at 94 °C for 3 min, followed by 40 cycles of 30-s denaturation at 94 °C, 30-s annealing at 54 °C and 2-min elongation at 72 °C; and a final extension hold at 72 °C for 7 min (Atopkin et al. 2017).

For electrophoresis, a 1% agarose (in TAE 1X-buffer) gel was used. Five µl of DNA PCR products were mixed with 1 µl of GelPilot® DNA Loading Dye (Qiagen, Hilden, Germany), premixed according to the manufacturer’s protocol and loaded for each sample. As reference, 6 µl of GelPilot® 1 kb Ladder (100) (Qiagen, Hilden, Germany), premixed according to manufacturer’s protocol, was added to the first well of the gel. Samples and ladder were dyed with GelRed® Nucleic Acid Gel Stain (Biotium, Inc. Fremont, California), according to the manufacturer’s protocol. Electrophoresis was run at 100 V, 130 mA and 50 W for an hour. Then, the gel was placed under ultraviolet transillumination and photographed. The samples showing bands were purified with a Qiagen QIAqick® PCR Purification Kit (Qiagen, Hilden, Germany) according to the manufacturer’s protocol.

For sequencing, we used the 3S (5′-GGTACCGGTGGATCACGTGGCTAGTG-3′) primer as mentioned by Atopkin et al. (2017), but the primer did not work even though the gel showed bands. Therefore, we used the PCR primers BD1 (5′-GTCGTAACAAGGTTTCCGTA-3′) and BD2 (5′-TATGCTTAA(G/A)TTCAGCGGGT-3′) for sequencing with a mixture of 10 µl of purified PCR product and 4 µl primer in a new labelled Eppendorf tube. Then, purified PCR products were sent to Microsynth Seqlab (Göttingen, Germany) for sequencing using the same amplification primers.

### Alignment and phylogenetic analysis

Contiguous sequences were edited manually and assembled using BioEdit 7.2. The resulting sequences were subjected to nucleotide BLAST searches to find the highest matching sequences available in the GenBank (http://www.ncbi.nlm.nih.gov/blast). A total of 22 sequences of ITS regions of other *Aspidogaster* spp. were downloaded from NCBI to infer the phylogeny of the worms obtained in the present study (Table 1). Phylogenetic analysis of the nucleotide sequences was performed using maximum likelihood (ML) methods. The model TIM2 + I was estimated as the best fitting for the data set using jModeltest v.2.1.10 software (Darriba et al., 2012) according to the Akaike information criterion (AIC). Phylogenetic relationship significance was estimated using a bootstrap analysis (Felsenstein 1985) with 100 replications. Phylogenetic trees were reconstructed with PhyML 3.1 software (Guindon and Gascuel 2003).

**Table 1:** List of available ITS rDNA sequences of *Aspidogaster* spp. with their host, location and references

Species	Host	Location	GenBank accession number	References
*A. chongqingensis* Wei et al., 2001	*Spinibarbus sinensis* (Cyprinidae, Teleostei)	Jialing River, Beibei, Chongqing, China	DQ345324	Chen et al. 2010
*A. limacoides* sensu Chen et al. (2010) Diesing, 1834 (Fig. 4)	*Coreius guickenoti* (Cyprinidae, Teleostei)	Jialing River, Beibei, Chongqing, China	DQ345319	Atopkin et al. 2017
*A. conchicola* Baer, 1827	*Mylopharyngodon piceus* (Cyprinidae, Teleostei)	Danjiangkou Reservoir, Danjiangkou, Hubei, China	DQ345317	Chen et al. 2010
*A. conchicola* Baer, 1827	*Mylopharyngodon piceus* (Cyprinidae, Teleostei)	Liangzi Lake, E’zhou, Hubei, China	DQ345318	Chen et al. 2010
*A. conchicola* Baer, 1827	*Colletopterum anatinum* (Unionidae, Bivalvia)	Tvertza River, Tver Region, European part of Russia	HE863962-HE863965	Atopkin et al. 2017
*A. conchicola* Baer, 1827	*Cristaria herc* (Unionidae, Bivalvia)	Khanka Lake, Primorskyi Region, Russia Far East	HE863958-HE863961	Atopkin et al. 2017
*A. ijimai* Kawamura, 1913	*Cyprinus carpio* (Cyprinidae, Teleostei)	Danjiangkou Reservoir, Danjiangkou, Hubei, China	DQ345320	Chen et al. 2010
*A. ijimai* Kawamura, 1913	*Cyprinus carpio* (Cyprinidae, Teleostei)	Jiangkou Reservoir, Xinyu, Jiangxi, China	DQ345321	Chen et al. 2010
*A. ijimai* Kawamura, 1913	*Cyprinus carpio* (Cyprinidae, Teleostei)	Niushan Lake, Wuhan, Hubei, China	DQ345322	Chen et al. 2010
*A. ijimai* Kawamura, 1913	*Cyprinus carpio* (Cyprinidae, Teleostei)	Jialing River, Beibei, Chongqing, China	DQ345323	Chen et al. 2010
*A. ijimai* Kawamura, 1913	*Cyprinus carpio* (Cyprinidae, Teleostei)	Khanka Lake, Primorskyi Region, Russia	HE863957	Atopkin et al. 2017
*A. ijimai* Kawamura, 1913	*Cyprinus carpio* (Cyprinidae, Teleostei)	Amur River, Nikolaevsk–na–Amure, Khabarovsk Region, Russia	HE863950-HE863957	Atopkin et al. 2017
*A. ijimai* Kawamura, 1913	*Cyprinus carpio* (Cyprinidae, Teleostei)	Amur River, near Khabarovsk, Khabarovsk Region Russia	HE866756	Atopkin et al. 2017
*A. ijimai* Kawamura, 1913	*Cyprinus carpio* (Cyprinidae, Teleostei)	Lake Biwa near Takashima city, Japan	MK387320-MK387330	Sokolov et al. 2019
*A. limacoides* sensu stricto Diesing, 1834	*Rutilus rutilus* (Cyprinidae, Teleostei)	Rybinsk Reservoir, Yaroslavl Region, European part of Russia	HE863966-HE863969	Atopkin et al. 2017
*A. limacoides* s. str. Diesing, 1834	*Blicca bjoerkna* (Cyprinidae, Teleostei)	Rybinsk Reservoir, Yaroslavl Region, European part of Russia	HE863970-HE863971	Atopkin et al. 2017
*Multicalyx elegans* (Olsson, 1869) (as outgroup)	*Callorhinchus milii* (Callorhinchidae, Chondrichthyes)	Australia, Hobart, Tasmania	DQ345325	Atopkin et al. 2017 from Gao et al. 2005 (unpublished)

## Results

*Aspidogaster limacoides* was found in the stomach and intestine of *Abramis brama*, *Rutilus rutilus* and *Scardinius erythrophthalmus*. This is the first study which confirms *A. brama* as a host of *A. limacoides* in Germany. The highest prevalence was recorded in *R. rutilus* (61.7%), while the lowest was in *A. brama* (2.9%). *Aspidogaster limacoides* was absent in *Perca fluviatilis*.

**Order: Aspidogastrida Dollfus, 1958**

**Family: Aspidogastridae Poche, 1907**

**Genus:** ***Aspidogaster*** **Baer, 1827**

**Species:** ***Aspidogaster limacoides*** **Diesing, 1834**

**Synonym:** ***A. donicum*** **Popoff, 1926**

**Fig. 1:**
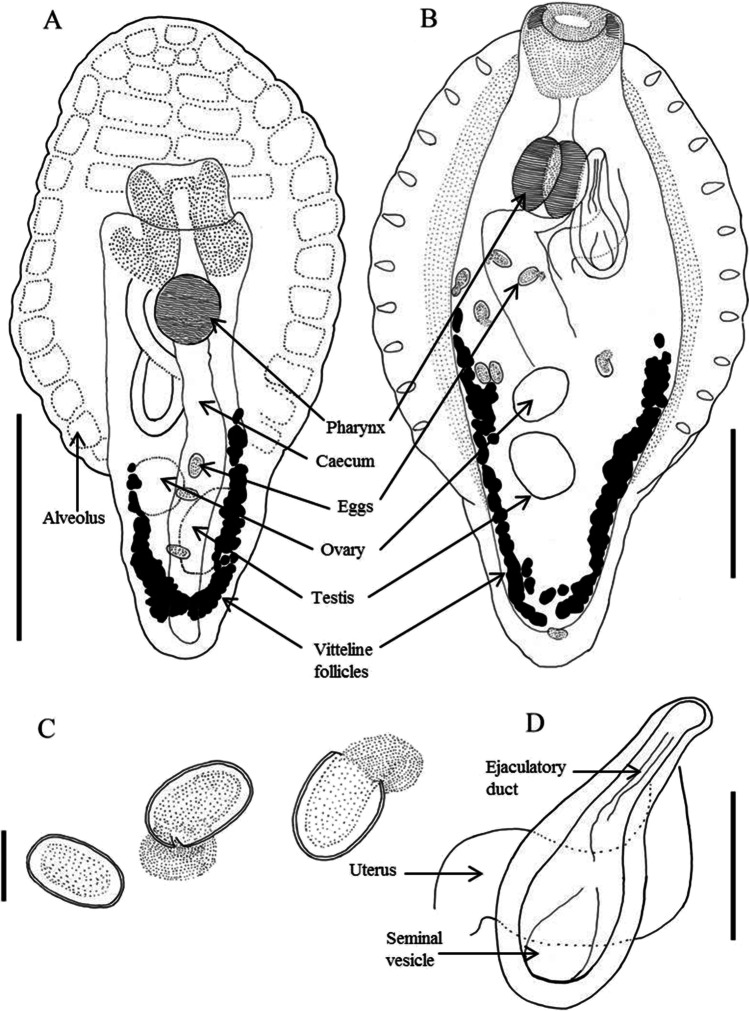
*Aspidogaster limacoides* line drawings from *Rutilus rutilus* from North Germany. **A** Dorsal view (scale bar = 500 µm); **B** ventral view (scale bar = 500 µm); **C** eggs (scale bar = 50 µm); **D** cirrus sac (scale bar = 200 µm)

**Fig. 2:**
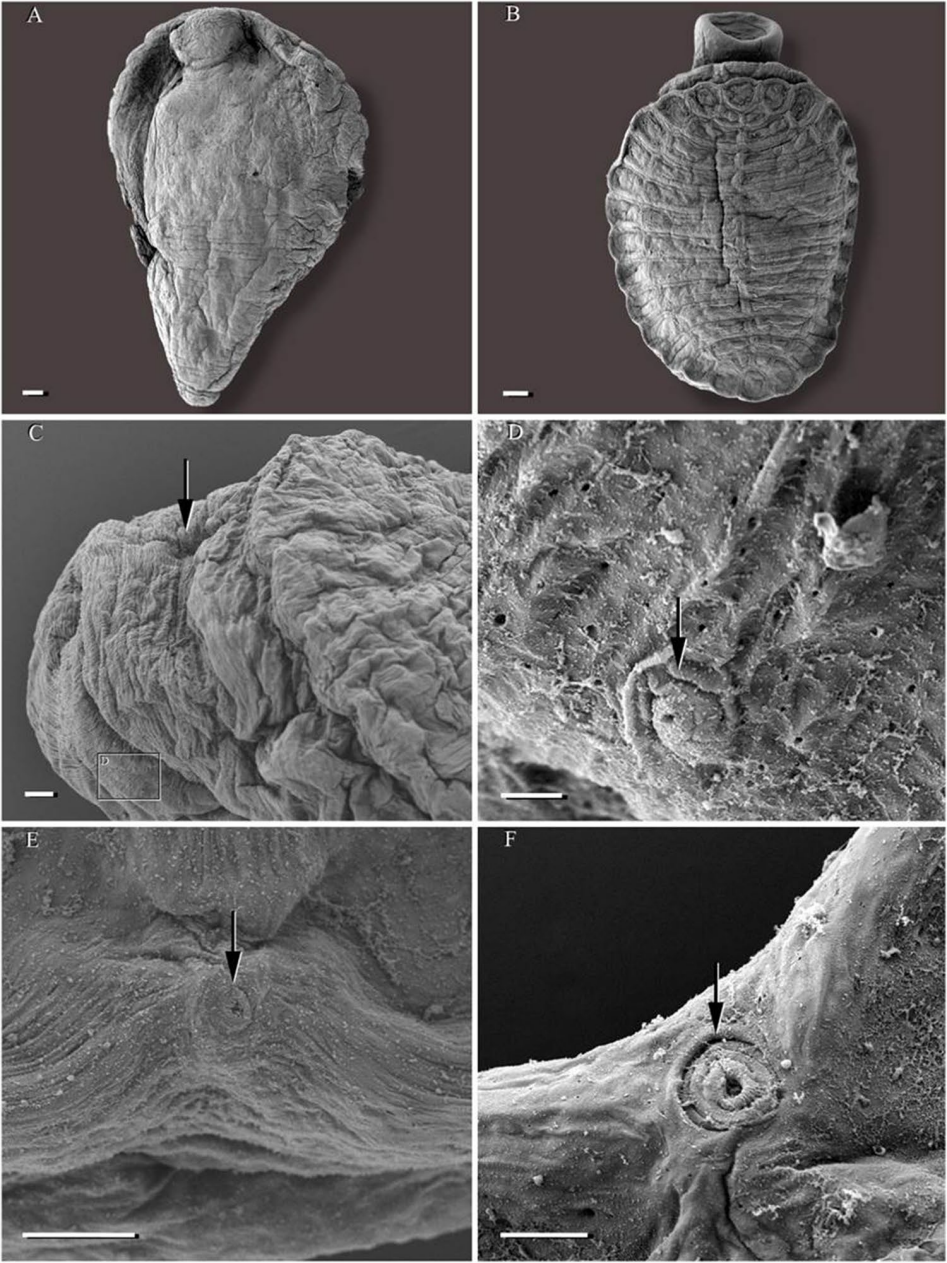
*Aspidogaster limcoides*, SEM: **A** dorsal view (scale bar = 100 µm); **B** ventral view with ventral disc (scale bar = 100 µm); **C** neck region, arrow showing depression of neck (scale bar = 20 µm), square **D** showing papillae-like structures posterior lateral to mouth; **D** posterior lateral papillae (arrow) (scale bar = 2 µm); **E** ventral disc, arrow showing marginal organ (scale bar = 20 µm); **F** ventral rim with marginal organ with terminal duct (arrow) (scale bar = 10 µm)

**Fig. 3:**
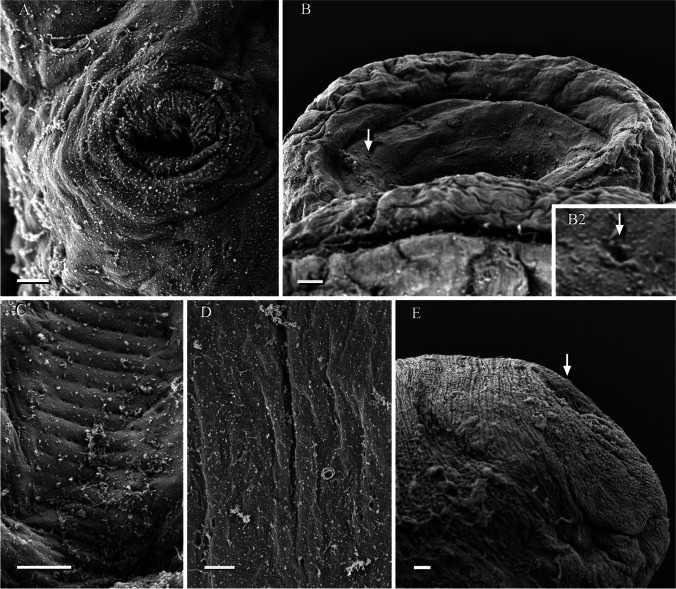
*Aspidogaster limcoides*, SEM: **A** marginal organ with terminal duct (scale bar = 2 µm); **B** and **B 2**: Arrow showing pits inside the mouth (scale bar = 20 µm); **C** Ventral disc showing pits on alveoli and septa (scale bar = 10 µm); **D** dorsal view, posterior body with pits (scale bar = 2 µm); **E** excretory pore (scale bar = 20 µm)

**Fig. 4:**
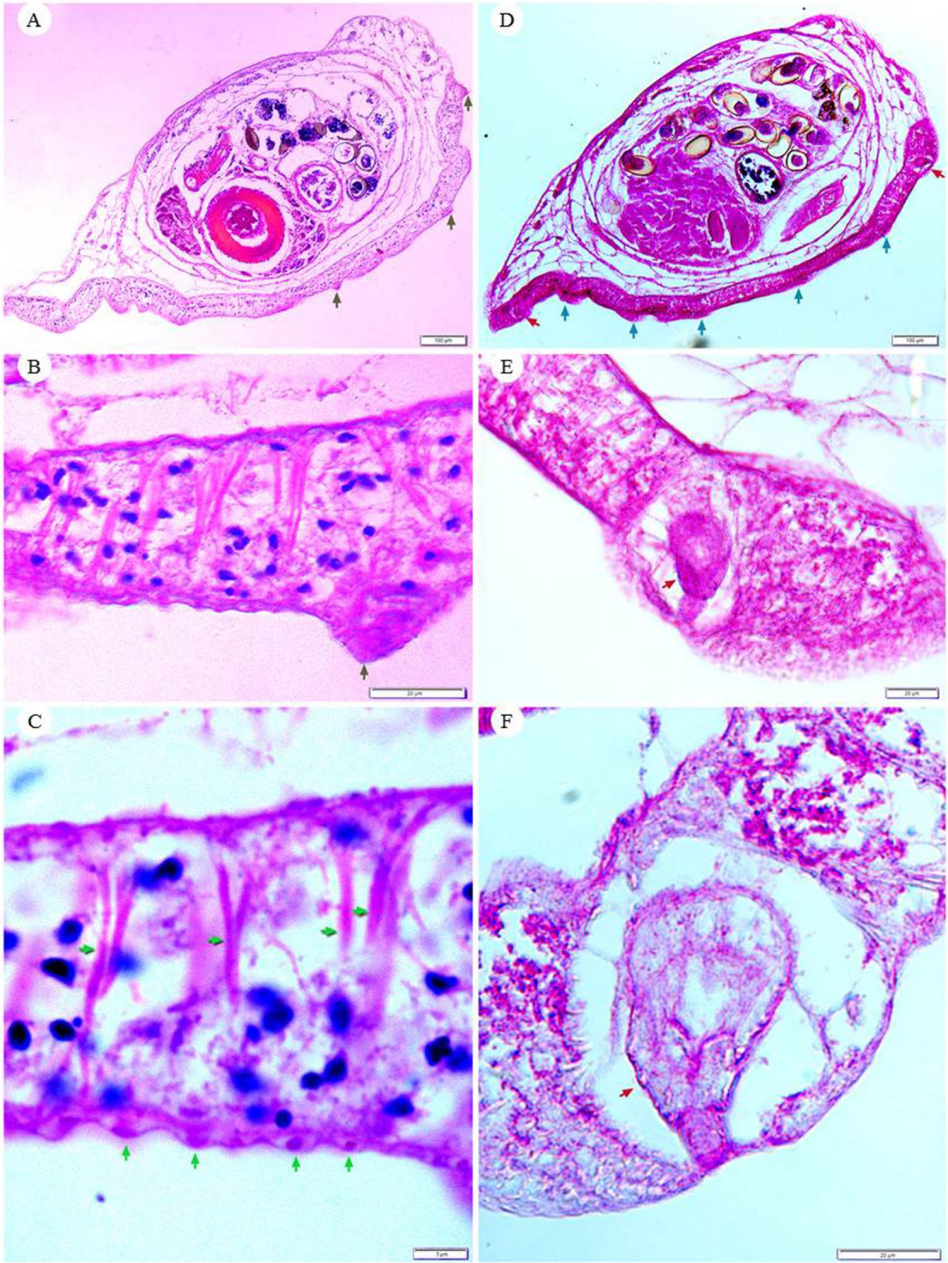
Cross-section of *Aspidogaster limacoides*, histology, from the level of the anterior edge of the ventral disc: **A** and **D** arrows (black and blue) showing marginal organs on ventral disc; **B**, **E** and **F** diagram of marginal organ (black and red arrows); **C** dorsoventral and longitudinal muscles (green arrows). Note: **A**–**C** H&E staining and **D**–**F** Alcian blue staining

**Description and measurements (in µm, Figs. 1–4):** Body elongated, bluntly rounded at the anterior and tapering towards the posterior end; widest at the level of the cirrus sac region (Fig. 1A, B). Body length (*n* = 13) 1,399–3,069 and width (*n* = 13) 470–1,082. Oral and ventral suckers absent. Mouth at anterior extremity, aperture circular or elongate (Fig. 1B). Oral aperture muscular. Buccal funnel surrounds mouth. Mouth cup-shaped (Fig. 2A, B). A few papillae-like structure posterolateral to the mouth (Fig. 2C, D). Ventral disc oval, bears 4 longitudinal rows each with 13–14 alveoli; 4 alveoli on each transverse row with individual alveolus at the anterior and posterior extremities; alveoli in median the rows transversely elongate, other alveoli more or less square or oval (Fig. 2B). Alveoli number varies from 54 to 58. Median alveoli more regularly arranged than the peripheral alveoli especially on the extreme ends of the periphery of the disc. Ventral disc covers most of body, often including mouth. Ventral disc length (*n* = 13) 1,261–1,923 and width (*n* = 13) 920–1,369. Marginal organs, as openings of exocrine, multicellular glands, pyriform, regular arranged on margins of both sides at junction of interalveolar septa of ventral adhesive disc (Figs. 2E and F, 3A, 4D–F). Opening of the terminal ducts of marginal organs at junction between transverse and longitudinal ridges on the rim of the ventral disc (Figs. 2E and F, 3A, 4D–F). Mouth connected to the pharynx via duct which gives funnel-shaped appearance to the mouth. Prepharynx length (*n* = 10) 183–367. Pharynx large, globular, strongly muscular. Pharynx length (*n* = 13) 203–320 and width (*n* = 13) 187–283. The intestine is simple, with single caecum, which reaches to the posterior end of the body (Fig. 1A). Caecum length (*n* = 1) 852. Presence of numerous pori (small openings) all over the body including inside and around the mouth, on neck, on fold between neck and ventral disc (including alveoli and on septa); dorsal, ventral, both lateral sides and posterior region (Figs. 2D, 3B-D). Excretory pore subterminal, towards more on the dorsal side (Fig. 3E).

Testis single, elongate, large and post-ovarian. Testis length (*n* = 5) 272–340 and width (*n* = 5) 170–239. Cirrus sac claviform, sinistral to pharynx, with doubled layered very thick muscular wall at proximal part, which becomes thinner at distal part. Cirrus sac length (*n* = 5) 402–537 and width (*n* = 5) 194–236. Internal seminal vesicle present, connected to pars prostatica, which opens into the ejaculatory duct. Genital pore not clearly visible, apparently opens at anterior level of pharynx.

Ovary single, globular and pretesticular. Ovary length (*n* = 2) 208–245 and width (*n* = 3) 141–170. The uterus is very long, fills posterior half of the body. Eggs are numerous, oval, often with opened operculum in distal part of the uterus. Egg length (*n* = 9) 72–93 and width (*n* = 9) 40–52.

Vitelline follicular, follicles globular, lateral fields beginning at equator, present on either side and confluent or nearly so at posterior end (Fig. 1A, B).

### Histology of the ventral disc

The ventral disc consists of four longitudinal rows of alveoli. In cross-section, the ventral disc consists of longitudinal muscles, marginal gland cells interspersed with dorsoventral muscles and a layer with more tightly arranged nuclei of the marginal gland cells (Fig. 4A–C). The marginal organ consists of the duct connected with the marginal gland cells, the ampulla with the secretion, a muscular papilla and the terminal duct and opening (Fig. 4D–F).

### Taxonomic summary

*Type hosts*: Not designated. First mentioned by Diesing. *Squalius cephalus* (L.) and *Leuciscus idus* (L.) (Diesing, 1834 (short version), 1835 (extended version)).

*Type locality*: South of Vienna, Austria (see Reimer 2002).

*Other hosts*: *Rutilus rutilus* (L.), *Scardinius erythrophthalmus* (L.) and *Abramis brama* (L.)

*Other locality*: Lake Tollensesee, Mecklenburg-Western Pomerania, Germany (53°30′26″N 13°12′41″E).

*Prevalence*: *Rutilus rutilus* 61.7%, *Scardinius erythrophthalmus* 7.7%, *Abramis brama* 2.9%

*Intensity (mean)*: *Rutilus rutilus*: 1–53 (9.3), *Scardinius erythrophthalmus*: 3 (3) and *Abramis brama*: 18 (18.0).

*Site of infection*: Mainly stomach and few specimens in intestine, post-mortem migration possible.

*Deposition of voucher specimens*: Natural History Museum Berlin (Museum für Naturkunde Berlin (ZMB), Germany); E.7645-E7647.

### Phylogenetic analyses

**Fig. 5:**
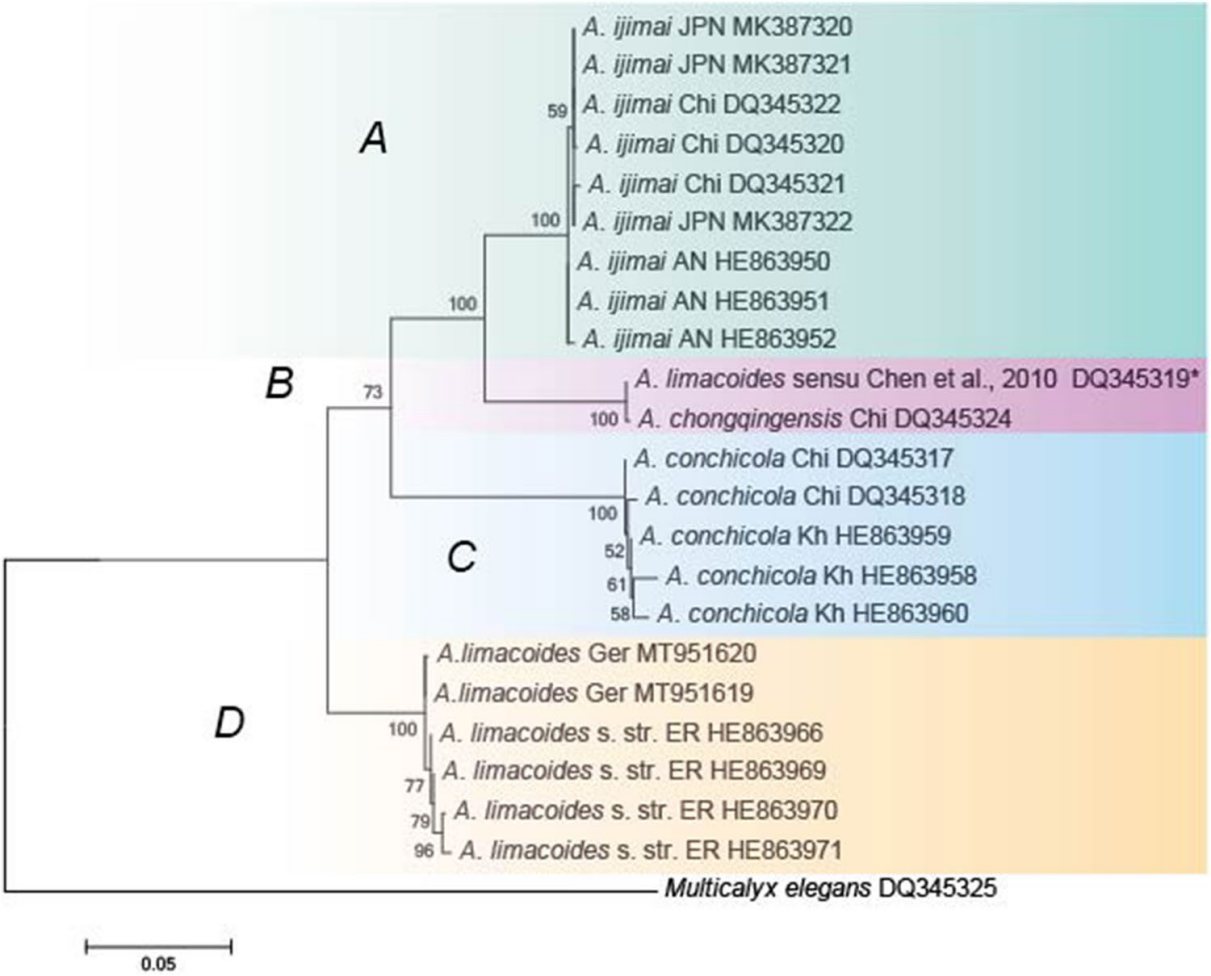
Phylogenetic tree based on analyses of ITS1-5.8S-ITS2 sequences of species belonging to the genus *Aspidogaster* using the maximum likelihood method of phylogenetic reconstruction with TIM2 + I model according to jModelTest software v 2.1.10. Nodal numbers give bootstrap statistical support for the analyses. AN, Amur River, Nikolaevsk-na-Amure; AK, Amur River, Khabarovsk; Kh, Khanka Lake; Chi, China; ER, European part of Russia; Ger, Germany; JPN, Japan. *Misidentified *A. chongqingensis*

Two contiguous sequences, 1,454 (accession number: MT951619) and 1,469 (accession number: MT951620) base pairs (bp) long, of the ITS1-5.8S-ITS2 region of rDNA were generated from adult worms isolated from *Rutilus rutilus* and *Abramis brama*. The newly obtained sequences varied by a single base. Our new sequence data showed 98.35% to 98.78% identity with ITS1-5.8S-ITS2 rDNA sequences of *A. limacoides* derived from *R. rutilus* sampled from Russia (accession codes: HE863971, HE863970, HE863969 and HE863966) using BLAST service. *A. ijimai* clade was subdivided according to geographical origin. This cluster formed a sister clade to *A. chongqingensis* with strong nodal support. Specimens of *A. conchicola* formed a distinct third clade. The fourth clade contained specimens of A. limacoides from German waters (present study) and the European part of Russia (Fig. 5).

## Discussion

The present specimens belong to the genus *Aspidogaster* Baer, 1827 due to the presence of a ventral disc with 4 longitudinal rows of alveoli, absence of head lobes, a single testis and a single blind caecum as described by Rohde (2002). *Aspidogaster limacoides* was firstly reported and described by Diesing from *Leuciscus cephalus* (L.) (now *Squalius cephalus*) and *L. idus* (L.) (Diesing 1834) and described in more detail with figures by Diesing (1835). Subsequently, Voeltzkow (1888) provided a further morphological description of Diesing’s type material. Additionally, this species was described by Bychowsky and Bychowsky (1934) from the Caspian Sea. Alves et al. (2015) summarized all host and locality records for this species. However, even though these authors recognized the publication of Reimer (2002) in a German magazine, they did not consider the record of *A*. *limacoides* from *R*. *rutilus* in the Weser River in North Rhine-Westphalia, Germany. Therefore, the present record is the second for *A. brama* worldwide (already known from Georgia, border between Europe and Asia) and the first in Germany, where only *R. rutilus* has been recognized as a host so far.

The prevalence and mean intensity vary greatly between these fish species. The highest prevalence of *A. limacoides* was reported in *R*. *rutilus* followed by *S*. *erythrophthalmus* and *A*. *brama*. However, the highest mean intensity was found in *A. brama*, suggesting also this cyprinid as a regular host. *Rutilus rutilus* and *S*. *erythrophthalmus* can be considered as the most common hosts for *A. limacoides*, and Zhokhov (2001) considered that the significant variations in the occurrence of *A. limacoides* in these fish species were mainly due to their different feeding behaviours. The author suggested that *A. limacoides* could be used as a feeding indicator in four different cyprinid fish species.

So far, within the genus, scanning electron microscopy has been used for only *A. ijimai* and *A. conchicola* (Gao et al. 2003). This is the first study which provides SEM pictures of *A. limacoides*. Topographical features such as the number and arrangement of alveoli and pits and the presence of papillae-like structures as well as the position of the excretory pore are unique for each of these species (Table 2). Yamaguti (1963) suggested that the number of alveoli is useful for the differentiation of *Aspidogaster* species, with *A. ijimai* having 42 alveoli, *A. limacoides* 50–74 and *A. conchicola* 60–174. This observation was later confirmed by several authors. Popiołek et al. (2007) quoted authors including Skrjabin (1952), Bauer (1987) and Bykhovskaya−Pavlovskaya et al. (1962) that the number of alveoli in *A. conchicola* and in *A. limacoides* is higher than 110 and fewer than 70, respectively. Based on the figure provide by Bychowsky and Bychowsky (1934), the number of alveoli ranged from 50 to 74 for *A. liacoides*. Similarly, Reimer (2002) observed 50 to 74 alveoli in *A. limacoides* from the river Weser, Northwest Germany, if considering 4 alveoli on each transverse row and two terminal alveoli at each end, but Reimer did not provided pictures. In the present study, the number of alveoli in *A. limacoides* varied from 54 to 58 based on SEM, and 58 and 62 alveoli have been reported for this species by Atopkin et al. (2017) and Popiołek et al. (2007), respectively. The number of alveoli in *A. ijimai* was 46 based on the figure provided by Lee et al. (2017) and Sokolov et al. (2019), whereas in *A. conchicola*, 114 alveoli were counted based on the figure provided by Atopkin et al. (2017) and 110 as reported by Bakker and Diegenbach (1974).

**Table 2:** Comparison of topographical structures of three species of *Aspidogaster* based on SEM

Characters	Species		
	*A. limacoides* Diesing 1834	*A. ijimai* Kawamura, 1913	*A. conchicola* Baer, 1827
Mouth	Cup shaped^a^	Cup shaped^b^	Cup shaped^b^
Depression on neck	Present^a^	Present^b^	Present^b^
Microridges	Not observed^a^	Present^b^	Absent^b^
Alveoli	54-58^a^		
	58^c^	42^c^	114^c^
	62^d^	46^gh^	110^i^
	50-74^ef^		
Arrangement of alveoli	One alveolus on each end and four on each row^ac^	One alveolus on each end, two alveoli with two rows and four on each row^cgh^	One alveolus on both ends, two alveoli with two rows and four on each row^ci^
Pits	Numerous^a^	Numerous^b^	Numerous^bj^
Marginal organs	Present^a^	Present^b^	Present^b^
Number of marginal organs if considering margninal organs present on each interalveolar septa	28-30^a^		
	26-38^e^	24^c^	60^c^
	30^cd^	26^gh^	
Papillae-like structures	Present and located posterior-lateral to the mouth^a^	Absent^b^	Absent^b^
Non-ciliated bulbous papillae	Absent^a^	Present and scattered over the surface^b^	Present and scattered over the surface^b^
Uniciliated sensory structures	Absent^a^	Absent^b^	Present^bj^
Excretory pore	Sub-terminal^a^	Terminal^b^	Terminal^b^

^a^Present study; ^b^Gao et al. (2003); ^c^Atopkin et al. (2017); ^d^Popiołek et al. (2007); ^e^Bychowsky and Bychowsky (1934); ^f^Reimer (2002); ^g^Lee et al. (2017); ^h^Sokolov et al. (2019); ^i^Bakker and Diegenbach (1974); ^j^Halton and Lyness (1971)

In addition to the number of alveoli, the arrangement of the alveoli in transverse rows on the ventral disc also differs between the *Aspidogaster* species, e.g. *A. conchicola* has one alveolus at each end, two transverse rows with two alveoli (one anterior and one posterior) and 4 alveoli on each remaining transverse row (Atopkin et al. 2017; Bakker and Diegenbach 1974). A similar pattern was observed in *A. ijimai* (Atopkin et al. 2017; Lee et al. 2017). However, the present study reveals that *A. limacoides* has 1 alveolus on each end and 4 alveoli in the remaining transverse rows, without 2 alveoli in transverse rows, in agreement with Atopkin et al. (2017) and Bychowsky and Bychowsky (1934). This suggests that also the arrangement of the alveoli on the ventral disc is a useful character to distinguish the species of *Aspidogaster*.

Numerous pits were found in *A. ijimai*, *A. conchicola* and *A. limacoides*, but microridges were found only on the neck region in the trough of folds in *A. ijimai* while uniciliated sensory structures were observed only in *A. conchicola* (Gao et al. 2003). In this study, few papillae-like structures were found only in the posterior lateral to the mouth. However, they are scattered all over the surface in *A. ijimai* and *A. conchicola* (Gao et al. 2003). There is also a considerable difference on the posterior end of *A. ijimai*, *A. conchicola* and *A. limacoides*. The excretory pore is terminal in the former two species but subterminal (dorsoterminal) in the latter species. On the other hand, a couple of morphological features are common to these three congeners, e.g. the presence of marginal organs with their terminal ducts on the rim of the ventral disc at the junction of the longitudinal and transverse ridges and a depression in the neck region (see Table 2). According to Huehner et al. (1989), these marginal organs are probably used to store and release secretions for extracorporeal digestion. The histology of the marginal organs in the present study demonstrates the secretory nature of these organs. They represent openings of the exocrine multicellular glands, with a duct connected with the marginal gland cells, an ampulla with secretion, a muscular papilla and a terminal duct with an opening. This morphology corresponds to the marginal organ described for other aspidogastreans, e.g. for *Lobatostoma manteri* Rohde, 1973 by Rohde (1973, 1994).

This study is the first SEM examination of *A. limacoides* and a first detailed study of this species from a northern location in Germany. In comparison to earlier SEM examination, it is evident that this technique provides additional significant insights into relevant topographical features of *Aspidogaster* species, especially the bulbous papillae, distribution of the pori and clear discrimination and arrangement of the alveoli, which are of taxonomic and systematic importance in this interesting group of parasites. Therefore, further SEM studies are needed for better species description and to differentiate between the species not only inside *Aspidogaster* but also between other aspidogastrean taxa.

## Code availability

Not applicable.

## Data Availability

All data published within this text.
